# Gos: a declarative library for interactive genomics visualization in Python

**DOI:** 10.1093/bioinformatics/btad050

**Published:** 2023-01-23

**Authors:** Trevor Manz, Sehi L’Yi, Nils Gehlenborg

**Affiliations:** Department of Biomedical Informatics, Harvard Medical School, Boston, MA 02115, USA; Department of Biomedical Informatics, Harvard Medical School, Boston, MA 02115, USA; Department of Biomedical Informatics, Harvard Medical School, Boston, MA 02115, USA

## Abstract

**Summary:**

Gos is a declarative Python library designed to create interactive multiscale visualizations of genomics and epigenomics data. It provides a consistent and simple interface to the flexible Gosling visualization grammar. Gos hides technical complexities involved with configuring web-based genome browsers and integrates seamlessly within computational notebooks environments to enable new interactive analysis workflows.

**Availability and implementation:**

Gos is released under the MIT License and available on the Python Package Index (PyPI). The source code is publicly available on GitHub (https://github.com/gosling-lang/gos), and documentation with examples can be found at https://gosling-lang.github.io/gos.

## 1 Introduction

Existing genomic visualization tools are tailored towards specific tasks and as such are limited in terms of expressiveness (Nusrat *et al.*, 2019). In contrast, the Gosling visualization grammar (L’Yi *et al.*, 2021) defines primitives to transform and map genomic datasets to visual properties, providing expressive building blocks to compose scalable and interactive genomic visualizations. Under the grammar-based methodology, visualizations are constructed from atomic elements rather than piecing together predefined templates like in most genome browsers (Buels *et al.*, 2016; Kent *et al.*, 2002; Kerpedjiev *et al.*, 2018; Thorvaldsdóttir *et al.*, 2013).

Although Gosling is extremely flexible, its implementation prioritizes readability and interoperability over improved ergonomics within a specific programming environment. This design decision hinders the use of Gosling as an exploratory tool during analysis since its detailed JSON representation may be cumbersome to construct via programming languages most familiar to computational biologists, and deployment or visualizing local datasets requires the administration of a web server. Software libraries may reduce these complexities for a specific audience by introducing further abstractions; however, maintenance poses substantial long-term challenges for developers to ensure compatibility and synchronization with the evolving grammar. Nevertheless, several efforts have been made to integrate traditional web-based genome browsers into interactive computational environments (Barrios and Prieto, 2017; De Jesus Martinez *et al.*, 2022; Hershberg *et al.*, 2021), though most are template-driven (i.e. limited customizability) and are either lacking in terms of interactivity or provide limited support for local datasets.

**Fig. 1.**
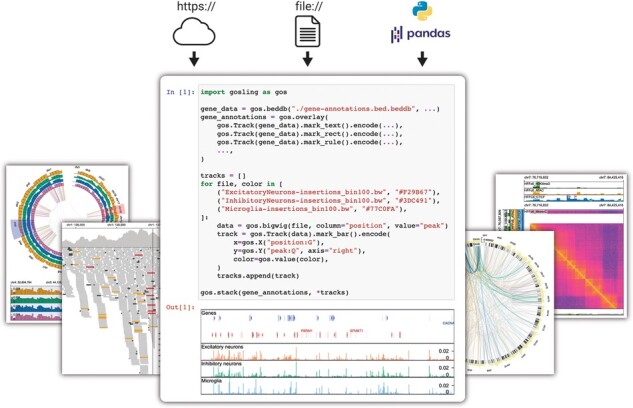
An overview of the Gos Python library. Gos supports remote and local genomics data files as well as in-memory data structures. Datasets are transformed and mapped to visual properties of marks via the Gos API to build custom interactive genomics visualizations within Python. For convenience, the field name and data type for an encoding may be specified with a simplified syntax (e.g. ‘peak: Q’ denotes the ‘peak’ variable with a quantitative data type). Visualizations are rendered directly in Jupyter, JupyterLab and Google Colab notebooks and can be exported as standalone HTML files

To address these limitations, we developed Gos—a Python library for computational biologists to create interactive genomics visualizations that can scale from whole-genome overviews to single nucleotides. Gos integrates seamlessly within interactive computational environments, containing utilities to host and display custom visualizations within Jupyter, JupyterLab and Google Colab notebooks (Fig. 1).

## 2 Implementation

The majority of the Gos Python API is automatically generated from the Gosling grammar. A custom code-generation program produces Python code for each primitive defined in the formal Gosling JSON schema. Complex visualizations are thus built via object-oriented programs rather than nesting and concatenating raw Python dictionaries. This implementation approach is inspired by and extends from Altair (VanderPlas *et al.*, 2018), a statistical visualization library based on Vega-Lite (Satyanarayan *et al.*, 2017). It guarantees that all visualizations are type-checked in complete concordance with the Gosling grammar and that Gos will remain consistent with the evolving schema over time.

The Gos library contains no visualization rendering code for increased modularity and is instead designed to export user-defined JSON data structures which adhere to the Gosling specification. The emitted JSON is easily rendered in a variety of web-based user interfaces with the Gosling JavaScript library. Gosling specifications defined via the Python API are rendered directly in the web-based user interfaces for Jupyter, JupyterLab or Google Colab notebooks. This HTML-based output overrides the text-based representation typically displayed for Python objects, presenting the user with an interactive graphic rather than the corresponding JSON definition.

Since rendering is decoupled from the Python API, users can control how the Gosling JSON is saved or displayed. Visual encodings are quickly explored with built-in rendering functionality, but alternative renderers may be selected for a specific use case if desired. For example, a visualization may be exported to a standalone HTML file or viewed via a custom Jupyter widget which allows controlling Gosling views from Python (e.g. programmatically navigating to genomic loci of interest).

## 3 Usage scenario

We provide a set of Jupyter notebooks (doi: 10.5281/zenodo.7321052) demonstrating how genomics visualizations may be created, combined and controlled with Gos. The clinvar.ipynb notebook contains a custom visualization to view human genetic variants from the ClinVar database (Landrum *et al.*, 2018) genome wide. This notebook uses the semantic zooming feature from Gosling to shift between aggregated bar and lollipop representations of variants, displaying detailed individual categorizations as well as summary distributions of clinical relevance depending on a zoom level. A predefined gene annotation track is used to provide context when navigating the viewer and demonstrates the reuse of common tracks across projects. Finally, we illustrate efficient navigation of the viewer to genomic regions of interest using a dropdown list of genes. This functionality showcases how users can further extend Gosling visualizations to fulfil specific tasks.

The getting-started.ipynb and navigation.ipynb notebooks introduce a basic concepts of the Gosling grammar through the declarative Python API, with sections detailing data-binding, creation of custom Tracks and Views (i.e. the main components for building Gosling genomics visualizations) and programmatic viewer navigation. The data-loading.ipynb notebook displays an identical visualization for a dataset hosted remotely, on the local filesystem, as well as a Pandas DataFrame, demonstrating the versatile data capabilities of our library.

## 4 Conclusion

Gos is a Python library for authoring interactive genomics visualizations via the Gosling grammar. Instead of editing deeply nested and repeated JSON data structures, users write simple Python programs which emit valid Gosling JSON specifications. Python scripts may therefore be repurposed to accommodate new data sources, offering a convenient utility to produce custom visualizations as a part of traditional bioinformatics pipelines.

Beyond this core functionality, Gos provides a unified framework to iteratively visualize genome-mapped data within interactive computational notebooks. Responsive genomics visualizations are woven between code and prose, and optional utilities host local and in-memory datasets without requiring users to exit the analysis environment or save intermediate results to disk. Gos connects flexible web-based genomics visualization with the larger scientific Python ecosystem, and we anticipate this integration can foster the use of numerical and machine learning libraries to actualize higher-level genomics visualization tools such as recommendation systems (Pandey *et al.*, 2022).

## Data Availability

No new data were generated or analysed in support of this research.

## References

[btad050-B1] Barrios D. , Prieto C. (2017) D3GB: an interactive genome browser for R, Python, and WordPress. J. Comput. Biol., 24, 447–449. 28437135 10.1089/cmb.2016.0213

[btad050-B2] Buels R. et al (2016) JBrowse: a dynamic web platform for genome visualization and analysis. Genome Biol., 17, 66. 27072794 10.1186/s13059-016-0924-1 PMC4830012

[btad050-B3] De Jesus Martinez T. et al (2023) JBrowse jupyter: a Python interface to JBrowse 2. *Bioinformatics. *10.1093/bioinformatics/btad032 PMC9887080 36648320

[btad050-B4] Hershberg E.A. et al (2021) JBrowseR: an R interface to the JBrowse 2 genome browser. Bioinformatics, 37, 3914–3915. 34196689 10.1093/bioinformatics/btab459 PMC8570803

[btad050-B5] Kent W.J. et al (2002) The human genome browser at UCSC. Genome Res., 12, 996–1006. 12045153 10.1101/gr.229102 PMC186604

[btad050-B6] Kerpedjiev P. et al (2018) HiGlass: web-based visual exploration and analysis of genome interaction maps. Genome Biol., 19, 125. 30143029 10.1186/s13059-018-1486-1 PMC6109259

[btad050-B7] Landrum M.J. et al (2018) ClinVar: improving access to variant interpretations and supporting evidence. Nucleic Acids Res., 46, D1062–D1067. 29165669 10.1093/nar/gkx1153 PMC5753237

[btad050-B8] L’Yi S. et al (2021) Gosling: a grammar-based toolkit for scalable and interactive genomics data visualization. *IEEE Trans. Vis. Comput. Graph.*, **28**, 140–150. 10.1109/TVCG.2021.3114876 PMC8826597 34596551

[btad050-B9] Nusrat S. et al (2019) Tasks, techniques, and tools for genomic data visualization. Comput. Graph. Forum, 38, 781–805. 31768085 10.1111/cgf.13727 PMC6876635

[btad050-B10] Pandey A. et al (2022) GenoREC: a recommendation system for interactive genomics data visualization. *IEEE Trans. Vis. Comput. Graph.*, **29**, 570–580. 10.1109/TVCG.2022.3209407 PMC10067538 36191105

[btad050-B11] Satyanarayan A. et al (2017) Vega-Lite: a grammar of interactive graphics. IEEE Trans. Vis. Comput. Graph., 23, 341–350. 27875150 10.1109/TVCG.2016.2599030

[btad050-B12] Thorvaldsdóttir H. et al (2013) Integrative genomics viewer (IGV): high-performance genomics data visualization and exploration. Brief. Bioinform., 14, 178–192. 22517427 10.1093/bib/bbs017 PMC3603213

[btad050-B13] VanderPlas J. et al (2018) Altair: interactive statistical visualizations for Python. J. Open Source Softw., 3, 1057.

